# Understanding the impact of spinal cord injury on the microbiota of healthy skin and pressure injuries

**DOI:** 10.1038/s41598-023-39519-2

**Published:** 2023-08-02

**Authors:** Reto Wettstein, Ezra Valido, Joel Buergin, Alexander Haumer, Nicole Speck, Simona Capossela, Jivko Stoyanov, Alessandro Bertolo

**Affiliations:** 1 SCI Population Biobanking and Translational Research Group, Swiss Paraplegic Research, Nottwil, Switzerland; 2 Department of Plastic, Reconstructive, Aesthetic and Hand Surgery, University Hospital of Basel, Basel, Switzerland; 3 Department of Health Sciences, University of Lucerne, Lucerne, Switzerland; 4 Institute of Social and Preventive Medicine, University of Bern, Bern, Switzerland; 5 Department of Orthopaedic Surgery, Bern Inselspital, University of Bern, Bern, Switzerland

**Keywords:** Clinical microbiology, Next-generation sequencing, Skin diseases

## Abstract

Pressure injuries (PI) are a common issue among individuals with spinal cord injury (SCI), especially in the sitting areas of the body. Considering the risk of infections occurring to PI during the wound healing process, the skin microbiome is likely to be a source of bacteria. We investigated the relationship between skin and PI microbiomes, and assessed any correlation with clinically relevant outcomes related to PI. Samples were isolated from SCI patients undergoing reconstructive surgery of PI, severity grades III and IV. DNA samples from skin and PI were analysed using 16S rRNA gene sequencing. Our results showed disparities in microbiome composition between skin and PI. The skin had lower diversity, while PI showed increased bacterial homogeneity as the severity grade progressed. The skin bacterial composition varied based on its location, influenced by *Cutibacterium*. Compositional differences were identified between PI grades III and IV, with clusters of bacteria colonizing PI, characterized by *Pseudomonas*, *Proteus* and *Peptoniphilus*. The skin and PI microbiomes were not affected by the level of the SCI. Our study highlights the differences in the microbiome of skin and PI in SCI patients. These findings could be used to target specific bacteria for PI treatment in clinical practice.

## Introduction

Pressure injuries (PI) are chronic wounds that are caused by multiple critical factors, some external—pressure, shearing forces, friction, and moisture—and other internal—malnutrition, anaemia and endothelial dysfunction^1^. The most significant factor contributing to PI is prolonged and unrelieved pressure over bony prominences^2^. The interruption of the blood flow leads to a shortage of oxygen and nutrients, and the accumulation of toxic by-products, ultimately results in tissue necrosis. Due to impaired mobility, lack of protective sensory perception and compromised wound healing of the denervated skin, PI are a common occurrence among individuals with spinal cord injury (SCI)^3^. PI are the second leading cause (16.5%) of unplanned hospitalisations among individuals with SCI (only behind urinary tract complications, 29.5%) and result in prolonged healthcare demands and costs^4^. The primary focus of PI treatments is to prevent further tissue breakdown by pressure relief and to obtain healthy and stable soft tissue that will resist prolonged pressure exposure in the sitting position^5^. The presence of necrotic tissue and clinical signs of infections can hinder the healing process and in addition are a contraindication for soft tissue reconstruction. Wound conditioning and debridements are performed to clean the injury, with granulation tissue formation as a sign of wound healing progression^6^. Despite successful management of post-operative infections, the microbiome of the skin and PI may contribute to the outcome of surgical management of bedsores. Currently, the role of the microbiome in the therapeutic outcome of surgical management of PI is under investigation^7–9^.

The PI microbiome, which refers to the microbial communities and their activities in the affected area, originates from the surrounding skin tissue ^7,10^ and can either aid or hinder healing by allowing colonization by commensal bacteria or pathogenic bacteria^11^. The skin is home to a variety of commensal bacteria, including *Actinobacteria*, *Firmicutes*, *Bacteroidetes*, and *Proteobacteria*, that play a role in maintaining skin health and preventing pathogen colonisation^12,13^. *Cutibacterium* (formerly *Propionibacterium*) *acnes* and *Staphylococcus epidermidis* are among the most commonly found bacteria on the skin*. Cutibacterium* reduces the skin pH by producing fatty acids^14^ while *Staphylococcus* releases glycerol which contributes to skin hydration^15^. The skin microflora is influenced by body location and moisture content (moisty, dry and sweaty)^16^, lifestyle, age and environment^13^. For instance, elderly individuals tend to have a reduced abundance of *Cutibacterium* spp. and the skin microbiome of bedridden elderly patients differs from that of age-matched ambulatory patients^7^.

Differing from intact skin, PI are characterised by the presence of aerobic or anaerobic bacteria, and fungi^17^. The most common microorganisms in PI are *Staphylococcus, Proteus, Pseudomonas* and *Enterococcus*^8,18^. In patients with SCI, the initial stages of PI are characterized by high relative abundance of *Staphylococcus*, *Anaerococcus* and *Finegoldia.* After four weeks, PI that worsen or remain unchanged clinically are dominated by *Proteus* and *Morganella*^9^. The density and type of these microorganisms are key factors^19^. Other factors such as microbial interaction, formation of a biofilm (microbial colonies embedded in a polymeric substrate), host immune response and skin tissue quality also determine the outcome of wound healing^20^. In severe cases of infected PI, individuals may develop complications such as cellulitis, abscess formation, bursitis, and osteomyelitis^21^.

The purpose of this study was to characterise the microbiome of PI in individuals with SCI undergoing reconstructive surgery for advanced stages of pelvic PI (grades III and IV) and to assess its impact on postoperative outcomes. By using next generation sequencing (NGS) of full-length universal 16S rRNA sequences, bacterial population compositions were analysed and compared with clinical data. Additionally, we analysed the impact of the level of paralysis on the skin microbiome, i.e. the presence or absence of sympathetic skin control and sensitivity.

## Results

### Participants' information

Bacterial DNA was isolated from swabs of skin and PI of 27 participants with traumatic SCI [all males; median age: 58 (range 29–78) years] who were undergoing reconstructive surgery, between June 2020 and September 2022 (Table 1). In 63% of the cases, the spinal cord injury was localized in the thoracic region and 67% of the patients showed no sensory or motor function in the sacral segments S4–S5 (ISNCSCI A). Additionally, 59% of the patients were classified as overweight or obese (BMI over 22.5 kg/m^2^ ^22^), and 22% were active smokers. Majority of the patients (37%) had no pre-existing medical conditions at the time of surgery, while the most common co-morbidities were hypertension (18%), peripheral vascular disease (11%) and renal disease (7%). Notably, 15% of the patients had multiple co-morbidities.

**Table 1:** Clinical data of patients included in the study.

Variable	Patients (n = 27)
Age (Years)	58 (52, 65)
Level of injury	
Cervical	8 (30%)
Thoracic	17 (63%)
Lumbar	2 (7%)
ISNCSCI score	
A	18 (67%)
B	5 (18%)
C	1 (4%)
D	3 (11%)
Body mass index (BMI)^†^	
< 22.5 kg/m^2^	11 (41%)
> 22.5 kg/m^2^	16 (59%)
Wound stage	
Grade III	14 (52%)
Grade IV	13 (48%)
Wound localization	
Ischial	18 (67%)
Sacral	3 (11%)
Trochanteric	6 (22%)
Area of the wound (cm^2^)	25 (15, 42)
Duration of wound	
< 1 month	13 (48%)
> 1 month	14 (52%)
Type of surgery	
Fasciocutaneous flap	26 (96%)
Myocutaneous flap	1 (4%)
Complications	
Yes, wound dehiscence/healing disorder (requires surgical intervention)	8 (30%)
No	19 (70%)
Underlying diseases	
Hypertension	5 (18%)
Peripheral vascular disease	3 (11%)
Renal disease	2 (7%)
Psoriasis (not on target region)	1 (4%)
Hyperparathyroidism	1 (4%)
Obstructive pulmonary disease	1 (4%)
None	10 (37%)
Multiple diseases	4 (15%)
Smoker	
Yes	6 (22%)
No	21 (78%)
Prealbumin units (g/L, n = 13)	0.21 (0.16, 028)
Albumin (g/L, n = 22)	36 (34, 38)
CRP (mg/L)	13 (4, 52)
Haematocrit (%)	34 (31, 40)
Creatinine (µmol/L, n = 26)	55 (46, 62)
Osteomyelitis	
Yes	5 (18%)
No	22 (82%)

Quantitative variables are expressed as median (Q1, Q3) and qualitative variables as absolute frequency (relative frequency in %).

^†^BMI value cut-offs for spinal cord injured people^22^.

In this study, all included patients had a severe form of pelvic PI: 52% grade III (no bone exposure) and 48% grade IV (with bone exposure). Osteomyelitis was present in 18% of the patients. The affected areas were the ischium (67%), sacrum (11%) and trochanter (22%). The median size of the injury was 25 cm^2^ (ranging from 6 to 77 cm^2^), and 52% of the injuries were present for more than one month. The majority of the reconstructive surgeries involved fasciocutaneous flaps (96%) and 30% of patients experienced wound dehiscence after the intervention.

Blood markers, such as albumin, prealbumin and creatinine, were investigated to assess the patients' nutritional status, particularly decreased protein intake. Results showed that the individuals in the study had levels of albumin (36 g/L) and prealbumin (0.21 g/L) within the normal reference ranges (albumin 34–54 g/L; prealbumin 0.15–0.36 g/L). However, the mean creatinine level (55 µmol/L) was below standard ranges (61.9–114.9 µmol/L). Additionally, 34% of participants had low haematocrit levels compared to the normal range of 40.7–50.3% for males without SCI. Furthermore, we observed slightly higher levels of C-reactive protein (CRP) levels in the SCI participants (in average 13 mg/L) compared to generally accepted normal range (below 8–10 mg/L).

### Microbial richness and diversity of skin and PI microbiota

The V1-V9 region of the 16S rRNA gene was amplified by PCR method and the MinION™ sequencing. Following adapter trimming, quality filtering and size selection, a median of 57,628 reads per sample (with an average read length of 1462 bp and 96.7% of reads classified) were retained for bacterial identification.

**Figure 1:**
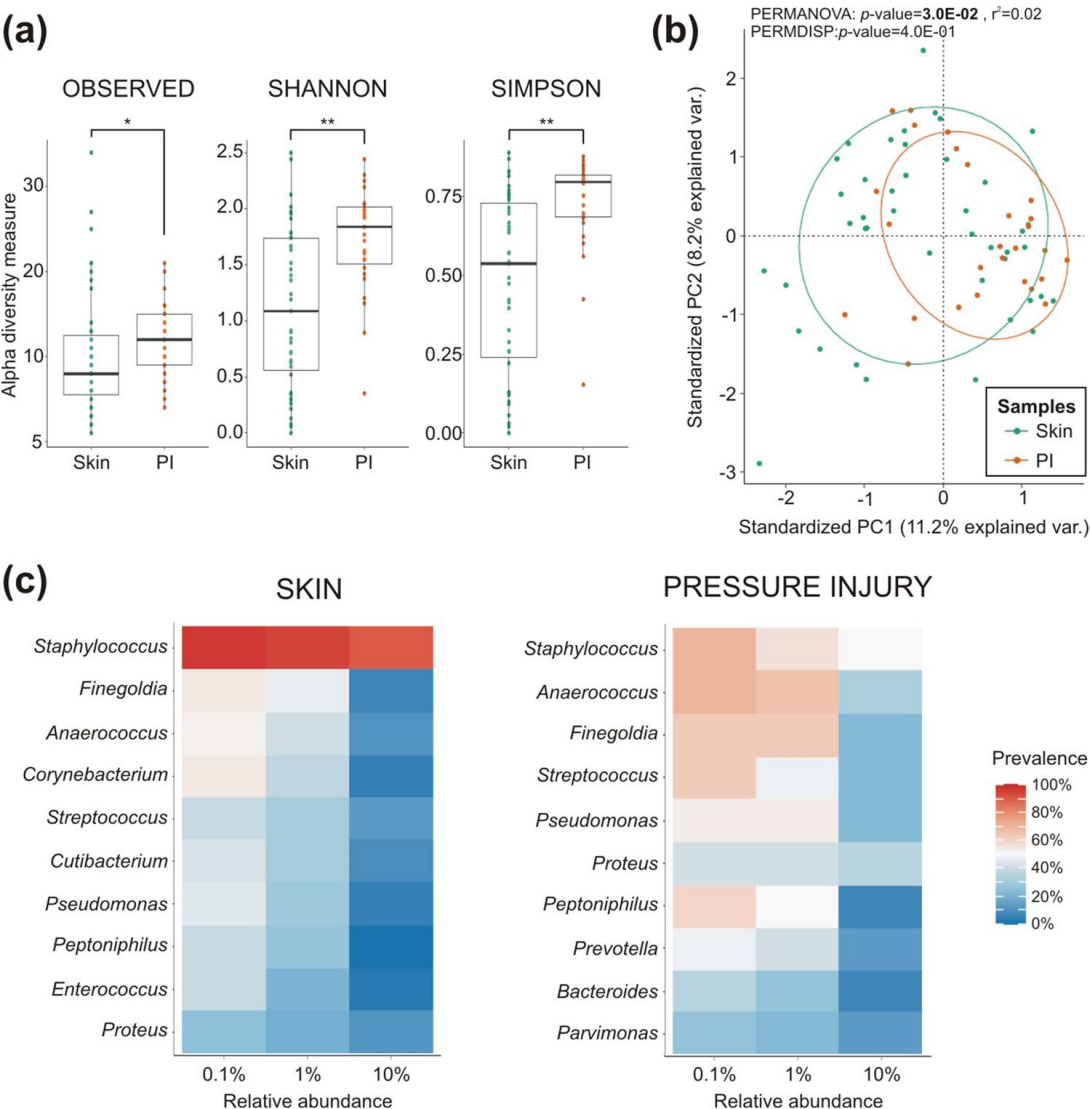
Comparison of bacterial communities isolated from skin (shoulder and pelvis samples) and PI in SCI patients. **(a)** Differences are shown by alpha diversity (number of observed genera, Shannon and Simpson indices) in the boxplots and **(b)** by beta diversity for the total population. Ellipses represent 95% confidence region of each group, skin in green and PI in orange (PC, principal component). **(c)** Ten most represented genera in skin and PI microbiomes. Genera are represented by their relative abundance (0.1%, 1% and 10%) and their prevalence across all samples analysed (skin, n = 43; PI, n = 27; *p < 0.05; **p < 0.01).

The sequencing identified a median of 8 genera in the intact skin (range 6–33) and with 12 genera in the PI (range 7–21; Fig. 1a). The PI showed a significantly higher level of microbial alpha diversity, as indicated by observed richness (*p*-value = 3.5E−02), Shannon (*p*-value = 3.8E−03) and Simpson (*p*-value = 4.3E−05) diversity indices compared to the skin microbiome (shoulder and pelvis together). This means that the PI samples contained a greater number of bacterial genera and a more even representation compared to the skin microbiome. In addition, a significant dissimilarity in the microbial composition (beta-diversity) was found between the skin and PI (*p*-value = 3.0E−02; Fig. 1b).

Skin and PI harboured different proportions of phyla, with *Firmicutes* and *Actinobacteria* being prevalent in the skin (in over 50% of the samples), while *Proteobacteria* was dominant in the PI. Among the *Firmicutes* phylum, *Staphylococcus*, *Finegoldia* and *Anaerococcus* were the most common in both the skin and PI, while *Streptococcus* (also *Firmicutes* phylum) was more prevalent in PI, and *Corynebacterium* (*Actinobacteria* phylum) was mainly found in the skin (Fig. 1c). The PI also showed increased prevalence of *Proteus* and *Pseudomonas* (*Proteobacteria* phylum) and *Peptoniphilus* (*Bacillota* phylum). The differential abundance analysis showed that *Staphylococcus*, *Corynebacterium*, Acinetobacter, *Cutibacterium* and *Brevibacterium* were significantly more abundant in the skin (Additional file 1: Table S1).

### The skin microbiota of SCI patients: differences between pelvis and shoulder

**Figure 2:**
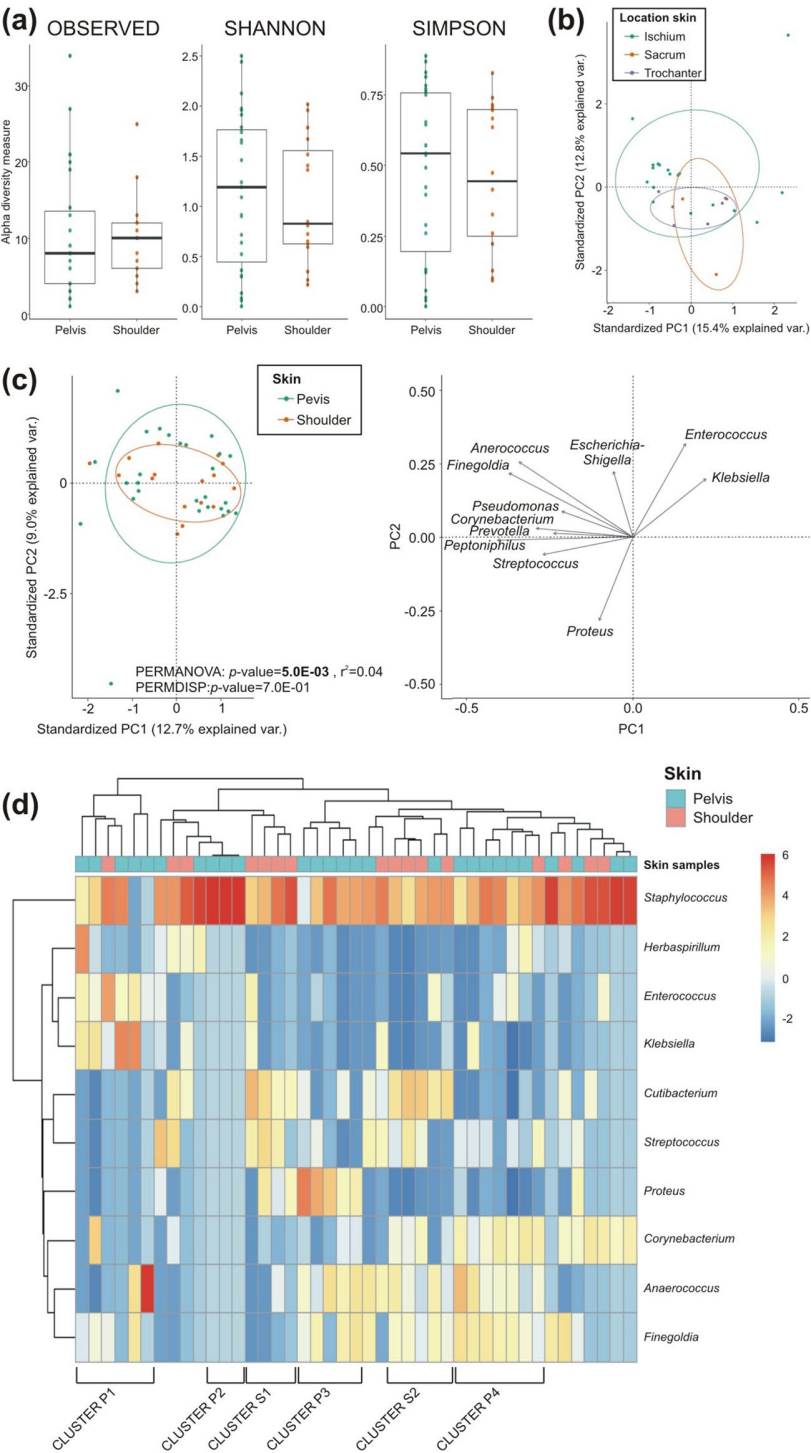
Differences in skin microbiomes of SCI patients between shoulder and pelvis samples. **(a)** Diversity between shoulder and pelvic samples were analysed by alpha diversity analysis (number of observed genera, Shannon and Simpson indices) in the boxplots. **(b)** Samples from different location within the pelvis region, namely ischium, sacrum and trochanter, were compared for beta-diversity in the biplot (ischium skin, n = 18; sacrum skin, n = 3; trochanteric skin, n = 6). **(c)** Beta diversity between pelvic and shoulder skin samples was analysed and arrows represent the influential loadings for principal component (PC)1 and PC2 of the principal coordinate analysis. **(d)** Hierarchical clustering of the centre-log ratio of the ten most relative abundant genera in pelvic and shoulder skin samples is shown (pelvic skin, n = 27; shoulder skin, n = 16; *p < 0.05; **p < 0.01).

We compared the bacterial DNA isolated from the skin near the PI, on the pelvis (within few centimetres distance; n = 27), with DNA isolated from the shoulder (far from the injury; n = 16). The median number of genera identified in the pelvic skin was 8 (range 2–33), while in the shoulder it was 10 (range 4–25; Fig. 2a). No significant differences were found in the microbial alpha diversity. When examining the pelvis, no differences were observed in the composition of the skin microbiome based on location on the pelvis (ischium, sacrum and trochanter; Fig. 2b). An increase in dispersion was noted moving closer to the anus—from the trochanter to the ischium—caused by contamination from faecal bacteria. However, the composition of the pelvic and shoulder microbiomes was significantly different (*p*-value = 5.0E−03, Fig. 2c). The presence of *Enterococcus* and *Klebsiella* was negatively correlated with the presence of *Proteus*.

Hierarchical clustering of the centre-log ratio of the ten most relative abundant genera revealed distinct clusters in the pelvis and shoulder skin samples (Fig. 2d). All samples were characterized by the dominance of *Staphylococcus*, except in two pelvic samples where *Anaerococcus* was dominant (Cluster P1). In this same cluster, a high prevalence of *Enterococcus* and *Klebsiella* was observed. In pelvic skin, threes additional clusters were identified: one was characterized by the unique presence of *Staphylococcus* (Cluster P2), while the other two were composed of *Anaerococcus* and *Finegoldia*, but differed in the presence of either *Proteus* (Cluster P3) or *Corynebacterium* (Cluster P4)*.* In the shoulder samples, two clusters were identified, both characterized by *Cutibacterium* and *Streptococcus* (Cluster S1), but differing in the presence of *Anaerococcus* and *Finegoldia* (Cluster S2). In the comparative analysis, only *Cutibacterium* and *Streptococcus* showed significantly higher abundance in the shoulder skin compared to the pelvic skin (Additional file 1: Table S1).

### PI microbiota: influence of wound severity grade and location on the pelvis

**Figure 3:**
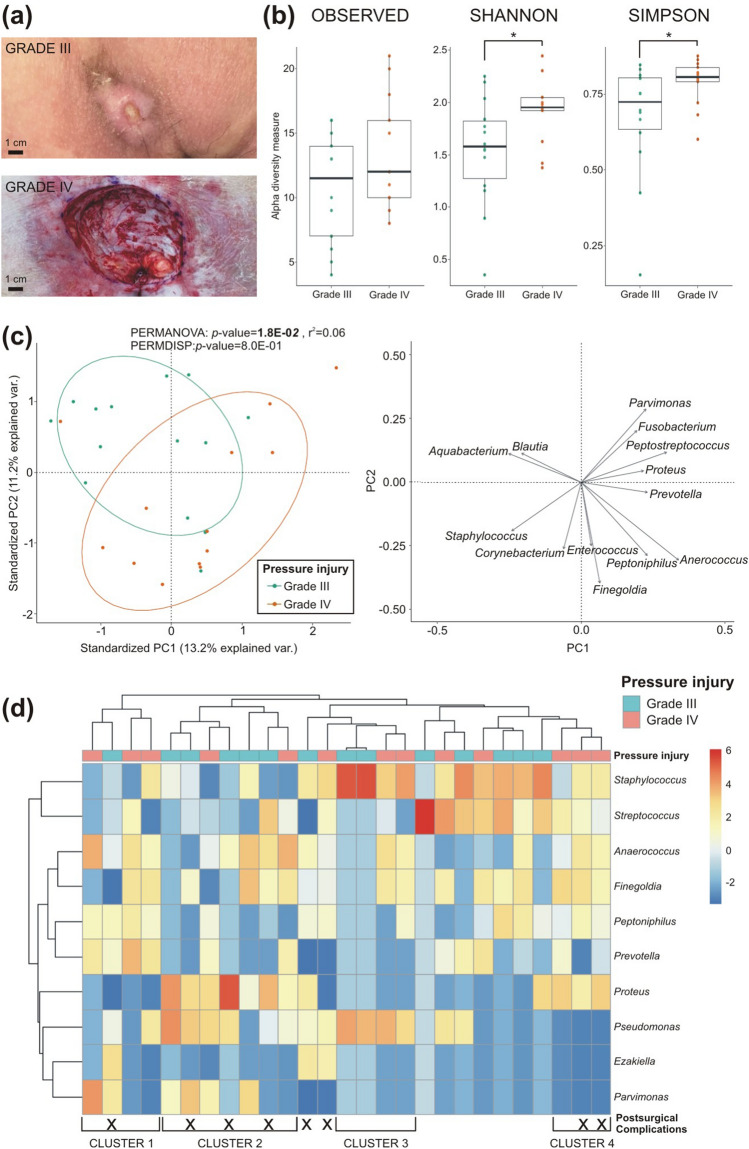
Differences in PI microbiomes of SCI patients between grade III and grade IV samples. **(a)** Representative pictures of grade III (above) and grade IV (below) pressure injuries included in this study. **(b)** Diversity between grade III and grade IV PI samples were analysed by alpha diversity analysis (number of observed genera, Shannon and Simpson indices) in the boxplots. **(c)** Beta diversity between grade III (green ellipse) and grade IV (orange ellipse) PI samples was analyses and arrows represent the influential loadings for principal component (PC)1 and PC2 of the principal coordinate analysis. **(d)** Hierarchical clustering of the centre-log ratio of the ten most relative abundant genera in grade III and grade IV PI samples is shown (grade III PI samples, n = 14; grade IV PI samples, n = 13; *p < 0.05; **p < 0.01).

The microbial communities in PI were found to differ based on severity grades (grade III vs grade IV; Fig. 3a). The analysis revealed a comparable median number of observed genera in grade III (12; range 4–16) and in grade IV (13; range 7–21; Fig. 3b). However, microbial alpha diversity as assessed by the Shannon (*p*-value = 3.8E−02) and Simpson (*p*-value = 4.3E−02) indices showed a significant increase in grade IV compared to grade III communities. The beta diversity between the two PI grades microbiomes was statistically significant (*p*-value = 1.8E−02, Fig. 3c). Higher presence of genera such as *Aquabacterium* and *Blautia* and lower presence of *Prevotella, Anaerococcus, Peptoniphilus* and *Finegoldia* were observed in grades III PI, as opposite to grade IV.

Hierarchical clustering of the ten most relative abundant genera revealed four distinct clusters, which differed in composition and the rate of postoperative complications (Fig. 3d). These four clusters were characterized by: (i) *Prevotella, Peptoniphilus* and *Anaerococcus* (Cluster 1; 1/4 cases with complications), (ii) *Pseudomonas* and *Proteus* (Cluster 2; 4/7 cases with complications), (iii) *Pseudomonas* and *Staphylococcus* (Cluster 3; 0/4 cases with complications) and (iv) *Staphylococcus*, *Streptococcus, Anaerococcus, Finegoldia, Peptoniphilus* and *Proteus* (Cluster 4; 2/3 cases with complications). Differential abundance analysis showed that *Campylobacter*, *Prevotella*, *Facklamia*, *Anaerococcus* and *Finegoldia* were more significantly abundant in grade IV PI (Additional file 1: Table S1).

The location of the PI on the ischium, sacrum or trochanter did not significantly affect the median number of observed genera (Additional file 1: Fig. S2a): ischium 12 (range 4 to 16), sacrum 17 (range 10 to 20) and trochanter 11 (range 6 to 21). The microbial composition of PI was found to be similar regardless of location, but the microbial composition dispersion was lower in the trochanter compared to the ischium and sacrum (Additional file 1: Fig. S2b).

### Comparison of standard microbial cultures and sequencing data of PI microbiome

**Figure 4:**
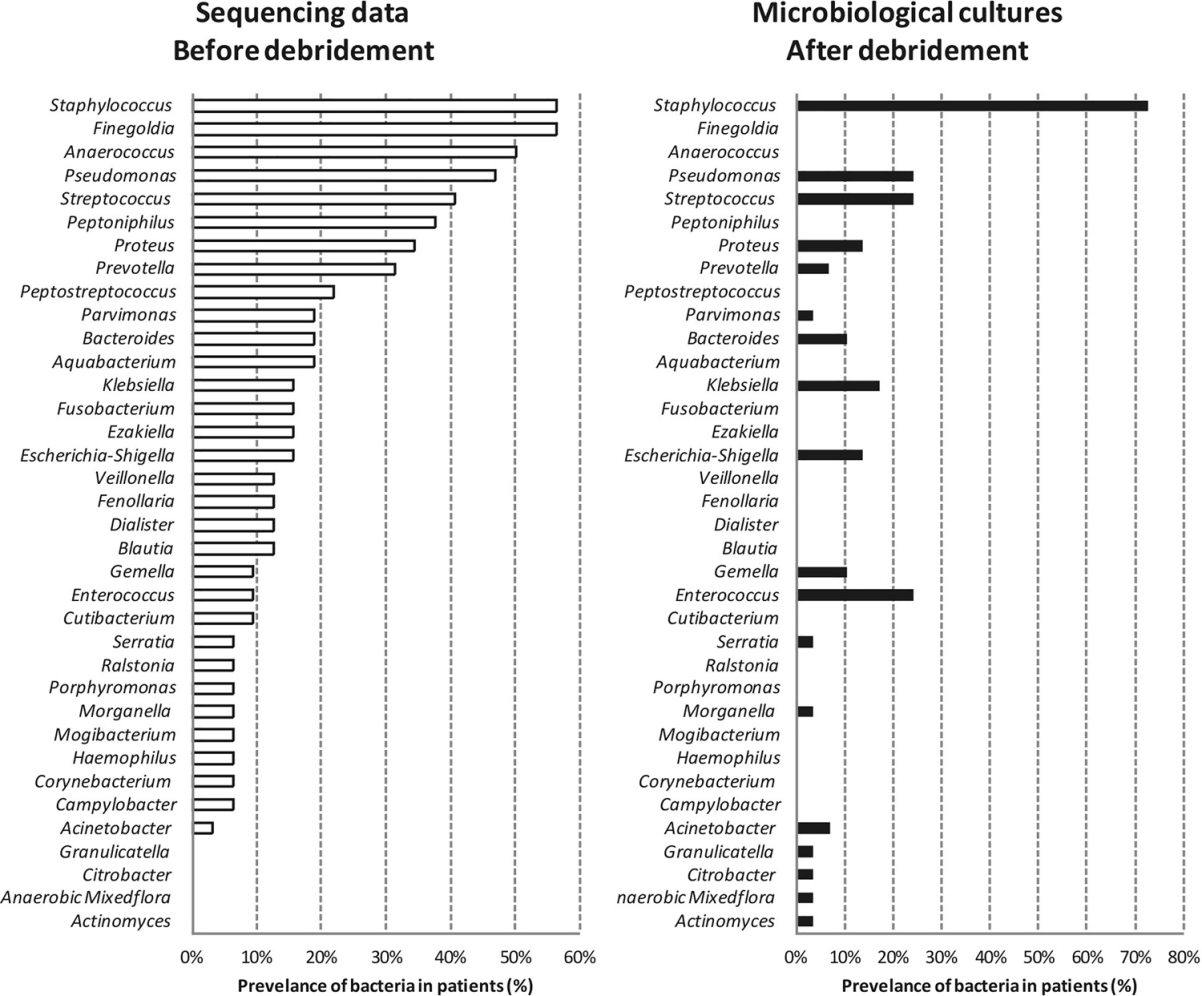
Comparison of results from sequencing data (swabs, on the left) and microbiological cultures (biopsies, on the right), obtained from pressure injuries (n = 27). The mean prevalence of bacteria across SCI patients is represented by each bar.

Results from both bacterial culture analysis using MALDI-TOF mass spectrometry and DNA sequencing from donors' PI were compared (Fig. 4). A bacterial genus was considered present in the PI with a relative sequencing abundance above 3%. The most prevalent genus was *Staphylococcus,* represented in 72% of the cultures and 56% of the sequenced DNA. *Klebsiella*, *Escherichia-Shigella* and *Gemella* were identified in the same proportions from both methodologies. However, bacterial cultures identified only 27% of the total genera isolated from the PI, missing the frequently detected by 16S sequencing anaerobes, such as *Finegoldia* (56%), *Anaerococcus* (50%), *Peptoniphilus* (38%) and *Peptostreptococcus* (22%)*.* Additionally, bacterial culture analysis underrepresented bacterial genera such as *Pseudomonas*, *Streptococcus*, *Proteus*, *Prevotella*, *Parvimonas* and *Bacteroides.* Conversely, bacterial cultures identified *Enterococcus* and *Acinetobacter* more frequently compared to DNA sequencing.

### Impact of spinal injury level on skin and PI microbiota

We examined the effect of SCI level on microbial diversity in skin and PI. The focus was on the influence of the loss of autonomic control of the skin, particularly the effect on sympathetic control over sweat glands and blood vessels (sympathetic skin responses) in cervical and thoraco-lumbar lesions (Additional file 1: Fig. S3a). The potential integrity of the sympathetic plexus provides more stable skin environment by regulating blood flow and sweating^23^. Indeed, compared to patients with thoraco-lumbar SCI, patients with cervical SCI showed a greater variation of microbial communities in pelvic (Additional file 1: Fig. S3b) and shoulder (Additional file 1: Fig. S3c) skin, even if non-statistically significant. *Klebsiella* associated negatively to *Streptococcus* in the skin pelvis and to *Peptoniphilus* and *Corynebacterium* in the shoulder. In the PI, the microbiome was not influenced by the lesion level either, but it was characterized by the absence of *Corynebacterium*, *Enterococcus,* and *Finegoldia* with cervical lesions (Additional file 1: Fig. S3d).

### Influence of surrounding skin on the PI microbiome

A comparison of skin and PI microbiomes was conducted to investigate their interdependence. Beta diversity analysis showed no difference in microbiome based on pelvis location, but a larger dispersion near the anus (Additional file 1: Fig. S4a). Further comparison of alpha and beta diversity between pelvic skin and PI in the ischium, sacrum and trochanter (Additional file 1: Fig. S4b–d) showed no significant difference in microbial composition, suggesting that the skin surrounding the PI has a direct role in shaping the PI microbiome. However, the microbial alpha diversity in ischial PI was found to be significantly higher for Shannon (*p*-value = 1.7E−02) and Simpson (*p*-value = 4.4E−03) diversity indices compared to the nearby skin microbiome communities.

### Postsurgical complications after PI surgery and analysis of microbiome

Eight patients (30%) experienced a complication with wound healing post-surgery (Additional file 1: Fig. S5a). The microbial community distribution varied between those with and without complications (*p*-value = 9.0E−03, Additional file 1: Fig. S5b) and *Ezakiella* was more abundant in post-surgical complication patients' PI (Additional file 1: Table S1). Additionally, patients with healing issues had larger wound size (38 cm^2^ vs. 20 cm^2^; *p* = 2.9E−02) and lower creatinine levels (38 µmol/L vs. 60 µmol/L; *p* = 7.0E−03) compared to those without wound dehiscence (Additional file 1: Table S2). Nevertheless, the influence of renal pathologies on creatinine levels cannot be ruled out. Furthermore, we found that PI microbial communities of patients with elevated CRP levels (above 10 mg/L) differed significantly from those with lower plasma CRP (below 10 mg/L) (*p*-value = 4.2E−02; Additional file 1: Fig. S5c).

## Discussion

In this study, we discovered that the microbiomes of PI in patients with SCI are composed of diverse and heterogenous mixture of both aerobic and anaerobic bacteria, forming a polymicrobial culture. The most prevalent PI bacteria belonged to seven genera: *Staphylococcus, Anaerococcus, Finegoldia, Streptococcus, Pseudomonas, Proteus and Peptoniphilus* (mostly belonging to the *Firmicutes* phylum*)* which have previously been also identified in prior research^9,23^. However, our results did not show a correlation between the PI microbiome and its anatomical location. Instead, our evidence suggests that the stage of tissue damage (grade III and IV) and postsurgical complications are linked to the characteristics of the microbial communities. Furthermore, we observed that microbiome in the PI was significantly associated to CRP concentration in plasma, applying a clinically relevant threshold of 10 mg/L. These observations lend additional support to the proposition that elevated bacterial counts and biofilm development could result in amplified wound inflammation and delayed healing. However, given the complex and multifaceted nature of pressure injury occurrence, we cannot conclusively establish causal relationships between CRP levels and the microbiome at this point.

We separated the PI microbiome in four distinct clusters, each with its own predominant bacteria. The cluster comprised of *Staphylococcus*, *Streptococcus, Anaerococcus, Finegoldia, Peptoniphilus* and *Proteus* showed a higher rate of complications (67% rate) compared to the other clusters (~ 20% average rate), though, the small number of participants in each group does not allow for any significant conclusion. The bacterial genus *Proteus* was associated to PI that developed wound dehiscence compared to other clusters. Previous studies have shown that the presence of *Proteus* is generally seen in worsening PI, while the bacterium *Corynebacterium* is seen to increase in relative abundance among those with improved PI ^9^. In this study, we have found *Corynebacterium* was significantly more abundant in skin compared to PI. Likewise, *Staphylococcus, Cutibacterium, Acinetobacter* and *Brevibacterium* were found to be differentially more abundant in skin, which are seen as skin microbiome commensals and believed to contribute to wound healing^24^.

In the context of wound healing, strict anaerobic bacteria can form symbiotic associations with aerobic or facultative anaerobic bacteria, which consume oxygen and create a favourable environment for the growth of strict anaerobic bacteria^25^. Previous studies have shown that strict anaerobic bacteria, such as those from the *Bacteroidales* family and *Streptococcus* can impede wound healing^26^. The use of molecular tools has revealed that anaerobic bacteria, which were previously under-represented by conventional bacterial cultures, are actually more prevalent in PI microbiota^9,24^. This study highlights the diversity of bacterial communities in PI, with different proportions of anaerobic bacteria, independently of debridement, which has been shown to not alter wound microbiome^27^. If a wound is not healing, the presence of certain types of strict anaerobic bacteria, such as *Finegoldia* and *Anaerococcus*, associated with *Enterobacteria*, may require frequent and thorough debridement. Oral antibiotic therapy has been shown to have limited effect on the PI microbiome diversity and composition, and is not recommended unless there are clear signs of infection, as it does not help to re-establish a beneficial equilibrium among the bacterial communities in the PI^9,28^. Thus, personalized diagnosis and treatment, which may involve regular molecular testing, in addition to conventional microbiological cultures, are necessary. However, molecular tests have limitations, such as not providing information on the viability of bacteria^29^. Customized and topical management can also help to fight against antibiotic resistance.

The composition of PI microbiome is largely impacted by the presence of commensal microbiota on the adjacent skin. These bacteria play a crucial role in maintaining a balance among the bacterial populations in the wound, preventing the growth of pathogens and controlling their severity^30^. Our investigation found that adjacent skin flora of the PI varied, depending on its location, but there was no significant difference in microbiome composition between the ischium, sacrum and trochanter areas. However, we observed an increased dispersion of microbiomes driven by the presence of faecal microorganisms near the anus. The comparison between the PI and nearby skin microbiomes showed no difference in microbial composition, reinforcing the idea that the PI microbiome is influenced by the surrounding skin. The skin microbiome can change based on other microclimate factors, such as pH, temperature, dryness, moisture and sebaceous gland activity^31^. Our investigation found no differences regarding the median number of genera between the skin in the shoulder and pelvic area, but difference in composition between the two microbiomes was significant. Dry skin areas, such as the shoulder, were significantly more populated by *Cutibacterium* and *Streptococcus*, while the pelvic area was contaminated by faecal bacteria.

The disruption of sympathetic nervous system innervation has minimal influence on the skin and pressure injury microbiota. Patients with an intact thoraco-lumbar sympathetic plexus have lower dispersion of bacteria in their microbiomes, compared to those with a SCI at the cervical level. We followed the autonomic control of the skin and their dermatomal innervations. In particular, we were interested in the loss of sympathetic control over the skin which affects cholinergic neurons that innervate sweat glands (sudomotor neurons) and adrenergic neurons innervating blood vessels and hair follicles (vasoconstrictor and pilomotor neurons)^32^. Indeed, we believe that the disparity results from the loss of control over the regulation of sweat glands (moisture of the skin) and vascularity (oxygenation), leading to a favourable environment for the growth of diverse bacteria.

As limitations, this study includes a small number of patients (n = 27) and a larger sample is needed to verify our findings. A metagenomic analysis, including fungi, viruses and multiresistant bacteria is also necessary to provide a more comprehensive view.

In summary, this study provides the opportunity to improve the management of PI. The development of molecular tests will be essential for the successful management of chronic wounds and personalized wound management, as it is challenging to culture and identify strict anaerobic bacteria. Prevention and clinical observation are critical components of PI management and play a key role in avoiding systemic infections.

## Methods

### Study population

Ethical approval for this study was obtained from the ethics committee of the northwest and central Switzerland (project ID: 2019-01062), and the study was performed in compliance with the Declaration of Helsinki**.** SCI Patients (n = 27) provided written informed consent upon enrolment. Male patients aged between 29 and 77 years old, with SCI and a grade III or IV PI (as defined by the European National Pressure Ulcer Advisory panel) were eligible for the study. The neurological impairment, level and completeness of the lesion were documented with the International Standard for Neurological Classification of SCI (ISNCSCI)^33^.

The clinical data of study participants included neurological assessment (level of injury, ISNCSCI score), nutritional status (Body Mass Index, serum prealbumin and albumin levels), underlying diseases (psoriasis, hypertension, peripheral vascular disease, diabetes, renal disease or urinary tract infection) and general health status (C-reactive protein (CRP), haematocrit and creatinine). PI severity grade (III and IV), duration of the PI from onset (less or more than one month), localization (ischium, sacrum and trochanter) and area of the wound (data from 65% of the patients) were assessed (see Appendix S1 in the Additional file 1 for further details about the surgical intervention).

### Sample collection and processing of samples

Swabs (Zymo) were collected from the wound bed (ischial, sacral and trochanteric regions) and from intact skin from 5 cm above the PI (pelvic skin) and on the shoulder, i.e. non-paralysed skin with intact sympathetic skin response (see Appendix S1 in the Additional file 1 for more details).

Bacterial samples were lysed using the ZymoBIOMICS DNA Miniprep kit (Zymo), according to the manufacturer’s instructions. In brief, vials containing swab heads were shaken with beads at 5000 rpm for 10 min, centrifuged briefly and DNA was extracted from supernatant. A negative control consisting in a swab soaked with in sterile 0.9% sodium chloride was included. The concentration of extracted DNA was not measured because it was in large proportion contaminated by human genomic DNA. Bacterial DNA samples were analysed by next generation sequencing of 16S ribosomal RNA (rRNA). The bacterial communities were amplified and barcoded according to adapted protocol by Oxford Nanopore Technologies (Protocol PCR barcoding amplicons, SQK-LSK109). Amplicon libraries were generated targeting the hypervariable regions 1–9 (V1–V9) region of the 16S rDNA, including a positive control—Microbial community standard (Zymo)—and a negative control processed equally to other samples during DNA extraction (Additional file 1: Fig. S1). Detailed methods are provided in Appendix S1 (Additional file 1). Output FASTQ files were uploaded to BugSeq (version 1.1, database version: RefSeq last Sep 18, 2022^34^) for 16S sequences classification^35,36^.

### Bacterial cultures

As standard clinical practice for microbiological analysis of the PI, biopsies were harvested from the wound bed (and/or exposed bone) using a Luer-forceps. In contrast to the swabs, the biopsies were harvested after disinfection and surgical debridement. The Matrix Associated Laser-Desorption-Ionization Time of Flight (MALDI-TOF) mass spectrometry was used to determine the bacterial species colonizing the PI. The bacterial cultures were performed in accordance with the standard clinical protocols used for analysis in hospitals.

### Statistical analysis

Results were analysed using R (version 4.2.2), at the genus levels. The alpha diversity was measured using the observed richness, Shannon, and Gini-Simpson indices. Statistical difference was reported using Wilcoxon Signed Rank Test with Benjamini–Hochberg adjusted *p*-values. The microbiome data was then transformed by centre-log ratio and the beta diversity was visualized using Principal Component Analysis (PCA). Beta diversity difference was tested using both permutational multivariate analysis of variance (PERMANOVA) and permutational multivariate analysis of dispersion (PERMDISP). Differential abundances were tested using analysis of composition of microbiomes with bias correction (ANCOM-BC2)^37^, linear models for differential abundance analysis (LinDA)^38^ and analysis of variance-like differential expression (ALDEx2)^39^. The differential abundance analysis was set with cut-offs of 0.1% relative abundance, with at least 10% prevalence. The accepted *p*-value for all analysis was ≤ 0.05.

## Supplementary Information

Supplementary Information.

## Data Availability

The datasets generated during the current study are available from the corresponding author on reasonable request.
