# Supplementary material for: Understanding the impact of spinal cord injury on the microbiota of healthy skin and pressure injuries
Source: Sci Rep. 2023 Aug 2;13:12540. doi: 10.1038/s41598-023-39519-2 (PMC10397227; doi:10.1038/s41598-023-39519-2)
Supplement: Supplementary file 1 — Supplementary Information. [file 41598_2023_39519_MOESM1_ESM.docx]

**The Impact of Spinal Cord Injury on the Microbiomes of Intact Skin and Pressure Injuries**

by Reto Wettstein^1,2†^, Ezra Valido^1,3†^, Joel Buergin^2^, Alexander Haumer^2^, Nicole Speck^2^, Simona Capossela^1^, Jivko Stoyanov^1,4^, Alessandro Bertolo^1,5*^

^1^ SCI Population Biobanking & Translational Research Group, Swiss Paraplegic Research, Nottwil, Switzerland.

^2^ Department of Plastic, Reconstructive, Aesthetic and Hand Surgery, University Hospital of Basel, Basel, Switzerland

^3^ Department of Health Sciences, University of Lucerne, Lucerne, Switzerland

^4^ Institute of Social and Preventive Medicine, University of Bern, Bern, Switzerland

^5^ Department of Orthopaedic Surgery, University of Bern, Bern Inselspital, Bern, Switzerland

† These authors have contributed equally to this work

* [alessandro.bertolo@paraplegie.ch](mailto:alessandro.bertolo@paraplegie.ch)

**Appendix S1** | **Supplementary** **Materials and Methods**

**Patients and sample collection**

Patients were recruited by multiple investigators (RW, JB, AH and NS) at the Swiss Paraplegic Centre (Nottwil, Switzerland) and sample collection was performed on the day of reconstructive flap surgery in the operation theatre before disinfection and debridement. Patients with non-traumatic SCI were excluded from the study. Samples were collected with a cotton swab soaked in sterile 0.9% sodium chloride and the area of interest – a 5x5-cm2 area – was firmly swabbed in a Z-stroke manner. The swab heads were placed into a vial containing DNA/RNA shield solution (Zymo, R1103) and stored at room temperature until bacterial DNA extraction.

**Clinical data from SCI patients**

The type of flap surgery performed and occurrence of any postoperative complications (delayed wound healing and wound dehiscence) were prospectively recorded. Patients received no antibiotic therapy before surgery. In absence of osteomyelitis, prophylactic antibiotic therapy was administered for 2 weeks after defect reconstruction. In the presence of osteomyelitis, the duration was 6 weeks and involved a specialized consultation in infectious diseases. After flap reconstruction, bed rest in an alternating pressure mattress lasted 4 weeks in a primary PI and in absence of osteomyelitis, whereas 6 weeks in a recurrent PI or in the presence of osteomyelitis. Participants were followed-up for complications until they were discharged from the hospital.

**Bacterial genomes sequencing**

Bacterial DNA was amplified using a set of 16S universal primers: forward primer 27F (5'- AGAGTTTGATCCTGGCTCAG-3') and reverse primer 1492R (5'-CGGTTACCTTGTTACGACTT-3'). Each primer was tagged to allow the barcoding using The PCR Barcoding Expansion 1-96 (Oxford Nanopore Technologies, EXP-PBC096). The tags used were 5’-TTTCTGTTGGTGCTGATATTGC-3’ for forward primers and 5’-ACTTGCCTGTCGCTCTATCTTC-3’ for reverse primers. 16S amplification reactions consisted in 2 µL of the primer mix (final concentration of 400 nM), in a mixture with 10.5 µL bacterial gDNA template (or 1 ng of DNA of the mock community) and 12.5 µL LongAmp® Hot Start Taq DNA Polymerase (New England Biolabs, M0534), in a final volume of 25 µL. Specific products were amplified by a PCR system (T3000 Thermocycle, Biometra), following these settings: 1 minute at 94°C for polymerase activation (1 cycle); 20 seconds at 94°C for denaturation, 30 seconds at 48°C for annealing and 90 seconds at 65°C for extension (30 amplification cycles); a final step of 3 min at 65°C. To assess possible contamination, each PCR reaction included a no template control (NTC) sample, which did not amplify (data not shown). Following each PCR round, The PCR fragments were purified by SPRIselect magnetic beads (Beckman Coulter, B23317). After purification, the concentration of DNA fragments was measured by Qubit dsDNA BR Assay Kit using Qubit 4.0 fluorimeter (Thermo Fisher Scientific, Q33238).

Afterward, the purified DNA from 16S amplification reactions was barcoded by PCR, in a mixture consisting of: 1 µL of the barcoding primer mix, 11.5 µL of 16S amplified DNA (equivalent to a total amount of 0.5 nM of DNA) and 12.5 µL LongAmp® Hot Start Taq DNA Polymerase, in a final volume of 25 µL. The PCR reaction was carried out following these settings: 180 seconds at 94°C for polymerase activation (1 cycle); 15 seconds at 94°C for denaturation, 15 seconds at 62°C for annealing and 100 seconds at 65°C for extension (12 amplification cycles); a final step of 3 min at 65°C. Barcoded DNA was then purified by SPRIselect magnetic beads and measured by Qubit dsDNA BR Assay Kit using Qubit.

At this stage, the different barcoded DNA were pooled together (final 1 μg of multiple barcoded DNA) and processed for end repair and dA-tailing using the NEBNext® Companion Module for Oxford Nanopore Technologies® Ligation Sequencing (New England Biolabs, E7180). After a purification step using SPRIselect magnetic beads, Adapter Bead Binding buffer was added to the DNA library. After the quality control and priming of the flowcell (Flow Cell Mk I, R9.4, FLO-MIN106D), the purified DNA library (at 50 fmol) was loaded and initiated a standard 12 hours sequencing protocol using MinION Mk1C device (Oxford Nanopore Technologies).

Basecalling was performed with Guppy (version 6.3.7) agent integrated in the EPI2ME software (version 5.2.13, Oxford Nanopore technologies), and FAST5 files were converted to FASTQ files. Barcodes were trimmed and sequences were filtered to include only those with a q-score ≥ 9. Output FASTQ files were uploaded to BugSeq (version 1.1, database version: RefSeq last Sep 18, 2022 [24](#_ENREF_24)) for 16S sequences classification.[25](#_ENREF_25),[26](#_ENREF_26) Reads shorter than 1000 bp and longer than 1850 bp were discarded.

**Figure S1** | **Taxonomic assignments of a mock community and negative control analysed by MinION sequencing.** The full length of the 16S rRNA gene (V1-V9 regions) was amplified from a pre-characterized mock community sample (positive control, ZymoBIOMICS Microbial Community Standard, D6300) comprising eight bacterial species and sequenced on the MinION platform. Over fifty thousand reads were aligned directly to the reference genome database of representative bacterial species. In the negative control, only two hundred copies of *Massilia* were detected. All reads were assigned to bacteria present in the positive control, without misclassified (reads assigned to bacteria not present in the mock community). The relative abundance (%) of each taxon is shown in brackets. Pearson correlation analysis was done to identify the correlation coefficient to determine the correlation strength between the two datasets.


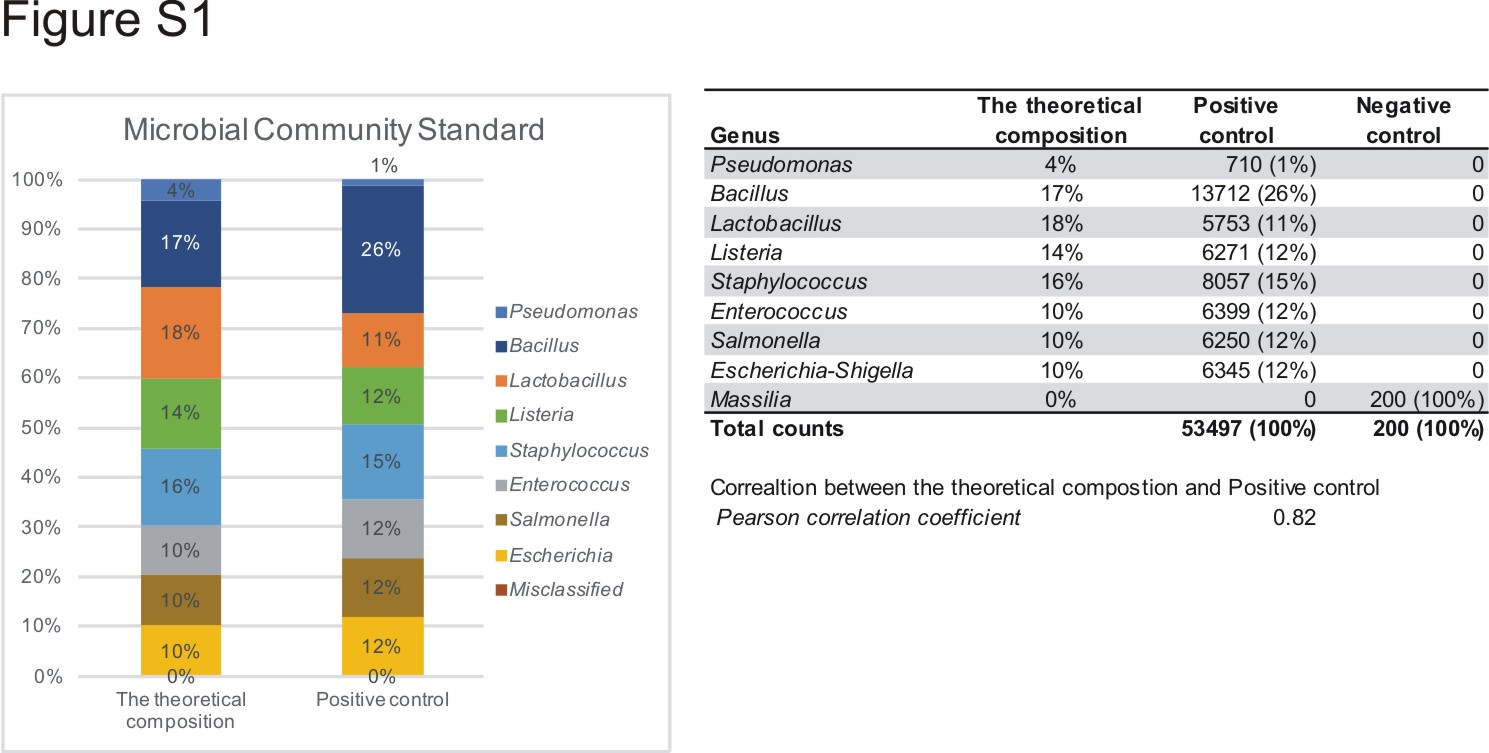


**Figure S2** | **Comparative analysis of bacterial diversity** **based on PI location on the patients' pelvis.** **(a)** Alpha diversity of samples isolated from PI located on ischium, sacrum and trochanter showed no differences in the observed richness, the Shannon index and the Simpsons diversity index, and **(b)** in principal component analysis plot to visualize beta diversity results**.**

**
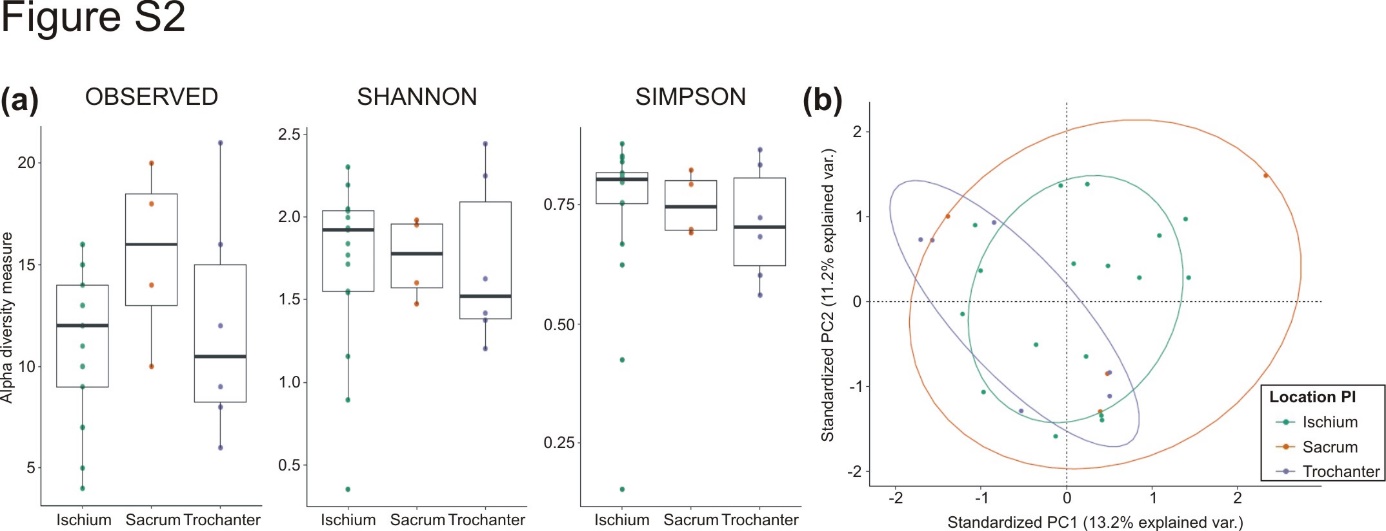
**

**Figure S3** | **The effect of SCI lesion level on the skin microbiome of the shoulder and pelvis areas, as well as the pressure injury. (a)** The diagram shows how the spinal nerves are distributed along the back according to dermatomes, and how they extend throughout the body (C=cervical, T=thoracic and L=lumbar regions). Beta diversity between cervical (green ellipse) and thoraco-lumbar (orange ellipse) regions samples was compared in pelvic skin **(b)**, shoulder skin **(c)** and PI **(d)**. Arrows represent the influential loadings for principal component (PC)1 and PC2 of the principal coordinate analysis. (In pelvis and PI: cervical samples, n=8; thoraco-lumbar samples, n=19; in the shoulder: cervical samples, n=5; thoraco-lumbar samples, n=11).

**
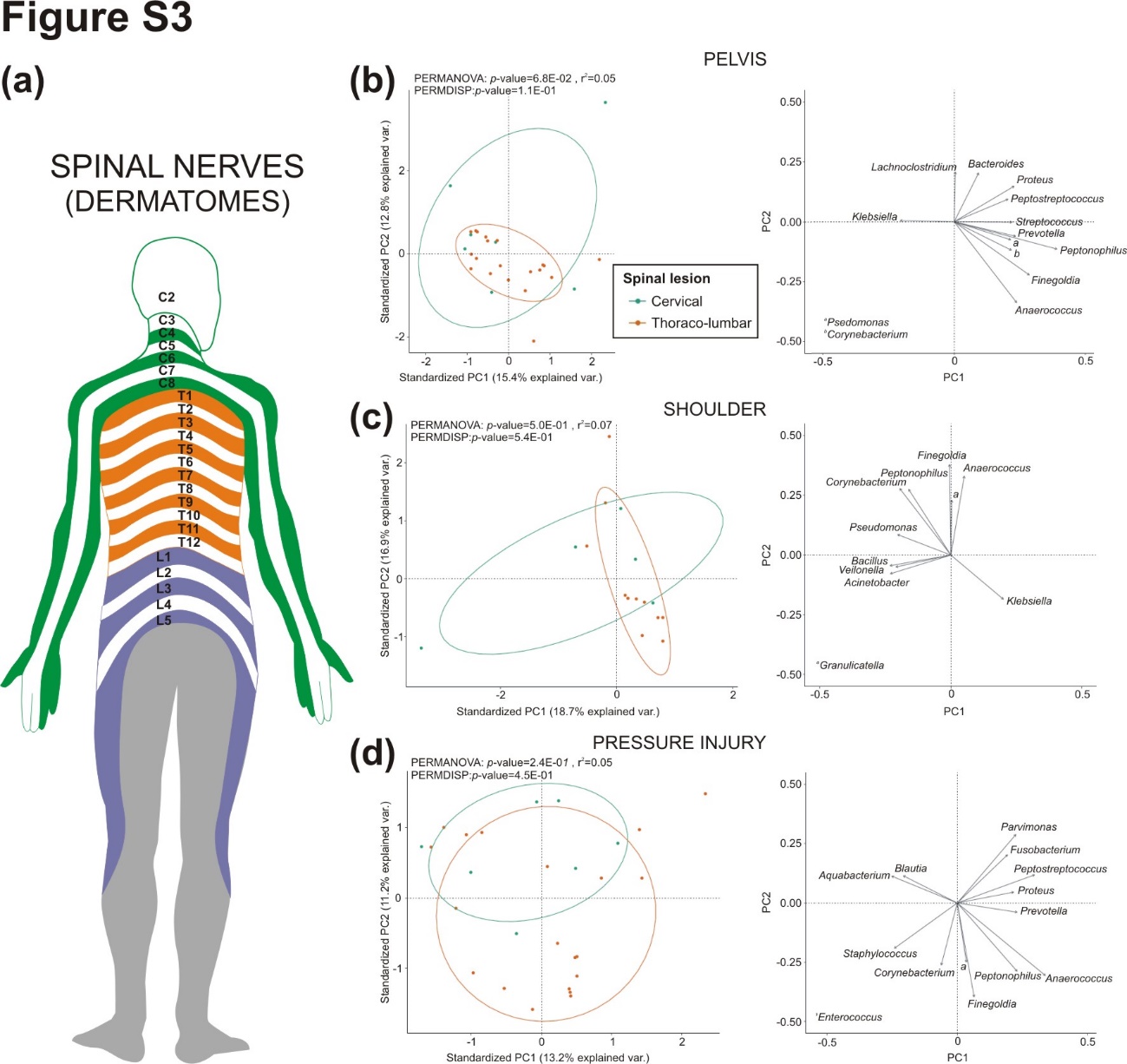
**

**Figure S4** | **Relationship between nearby skin and pressure injury microbiomes. (a)** Samples (both skin and PI) from ischium (green ellipse), sacrum (orange ellipse) and trochanteric (violet ellipse) regions were compared for beta-diversity (ischium skin, n=18; sacrum skin, n=3; trochanteric skin, n=6). Each region, namely ischium **(b)**, sacrum **(c)** and trochanter **(d)**, was then further analysed for alpha- and beta-diversity based on relative differences between skin (green ellipse) and PI (orange ellipse).

**
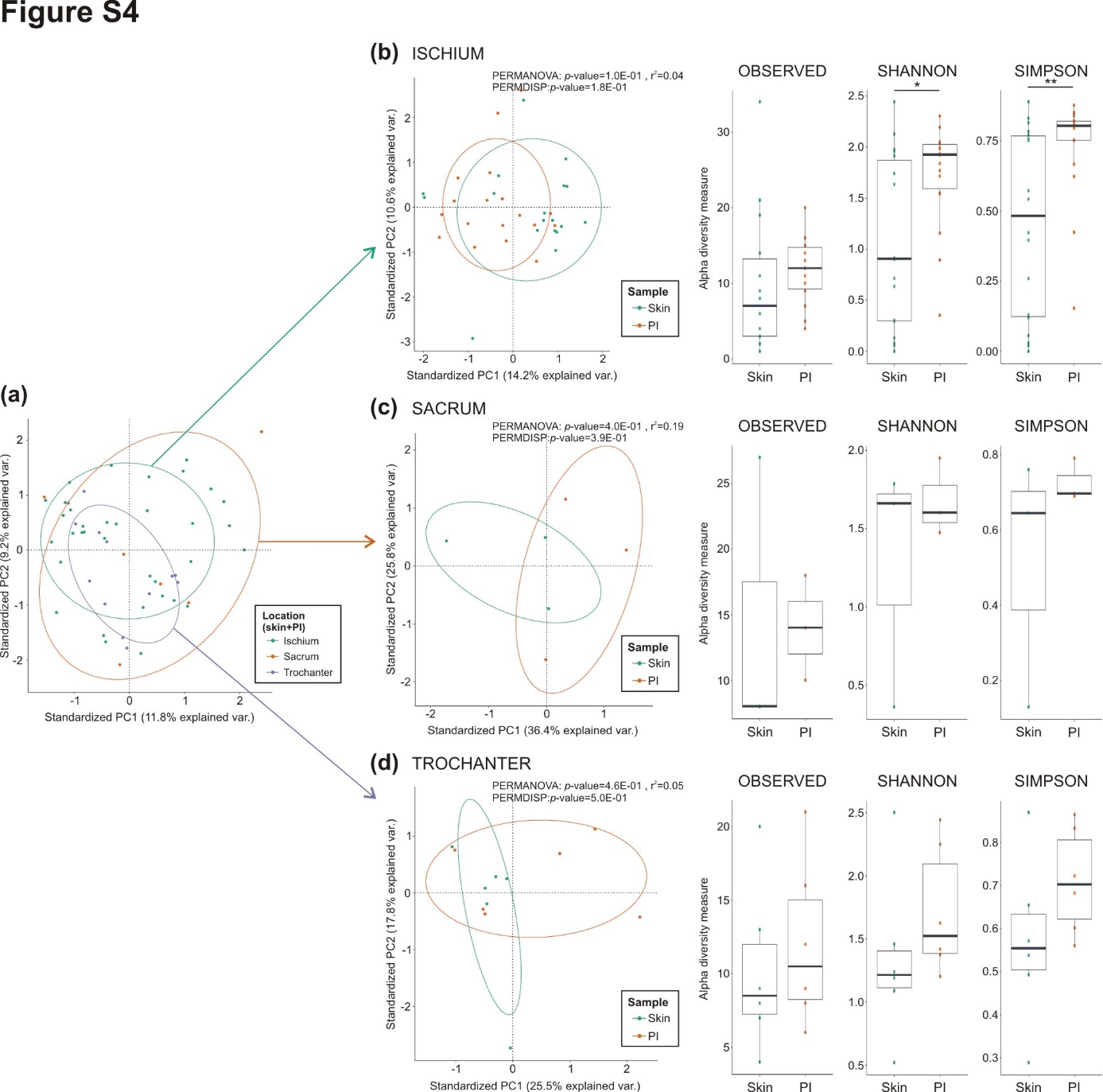
**

**Figure S5** | **Analysis of pressure injury microbiomes based on the occurrence of postsurgical complications. (a)** Representative pictures of typical healing (left) and wound dehiscence (right) in pressure injuries after surgery. **(b)** A comparison of beta diversity between PI that developed postsurgical complications (orange ellipse, n=8) and those who did not (green ellipse, n=19), and **(c)** based on the plasma level of C-reactive protein (CRP) above (green ellipse, n=15) or below (orange ellipse, n=12) the concentration of 10 mg/L.

**
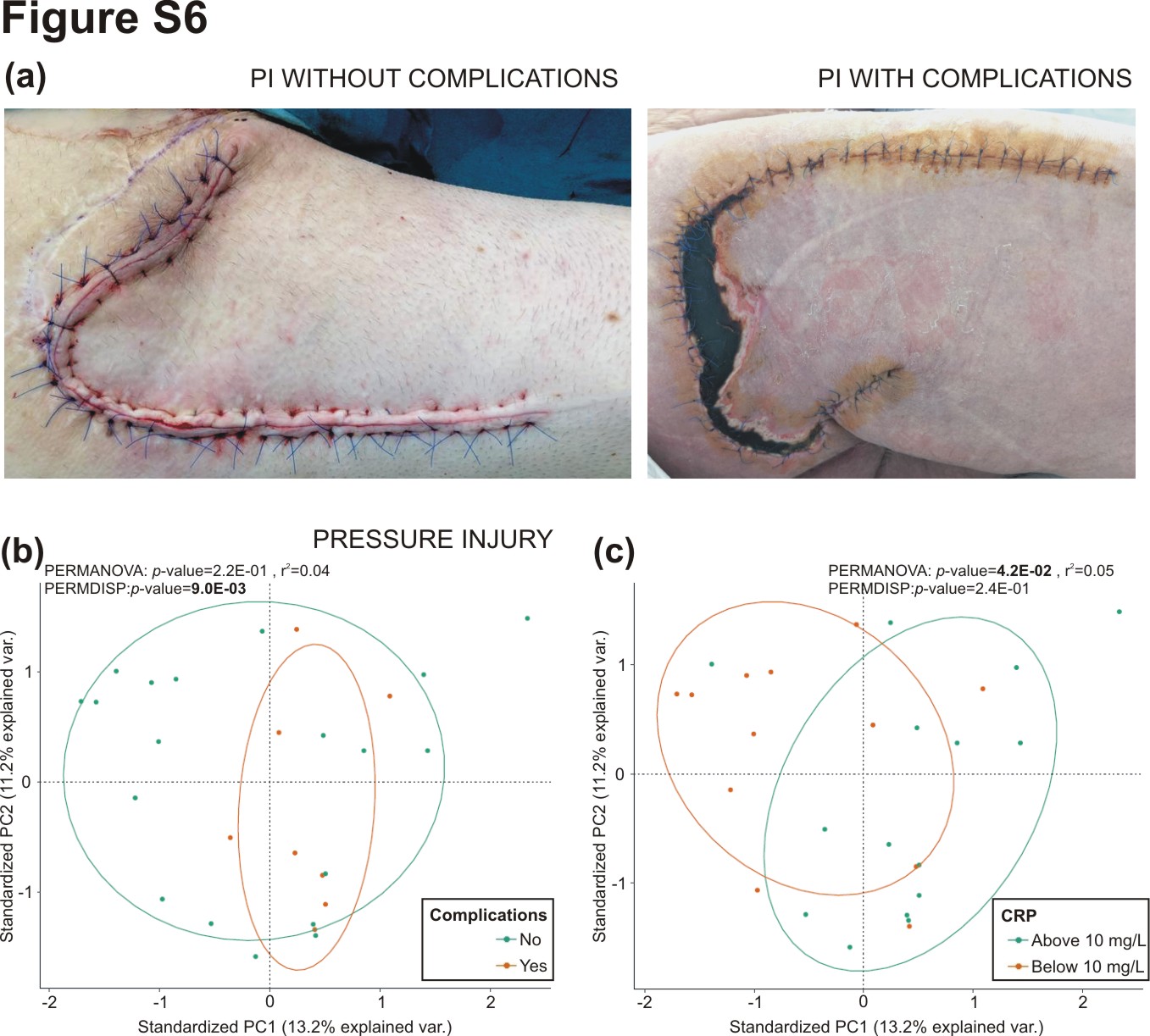
**

**Table S1** | Differentially abundant bacterial genera from the skin and PI microbiome of SCI patients.

| **Variables**  **(N)** | | **Taxonomic Group** | **Standardized Effect Size (log_2_[fold change])** | | |
| --- | --- | --- | --- | --- | --- |
|  |  | **Genus** | **LinDA** | **ALDEx2** | **ANCOM-BC2** |
| **Tissue (54)** | |  |  |  |  |
| Skin | PI | *Staphylococcus* | 0.82** | 0.62** | 0.67** |
| **↑** | **↓** | *Corynebacterium* | 0.60** | 0.50* |  |
|  |  | *Acinetobacter* | 0.50** |  |  |
|  |  | *Cutibacterium* | 0.47** |  |  |
|  |  | *Brevibacterium* | 0.44** |  |  |
| **Skin (43)** | |  |  |  |  |
| Shoulder | Pelvis | *Cutibacterium* | 0.91** | 1.14** | 0.56** |
| **↑** | **↓** | *Streptococcus* | 0.44* |  |  |
| **Pressure injury (27)** | |  |  |  |  |
| Grade IV | Grade III | *Campylobacter* | 0.64* |  |  |
| **↑** | **↓** | *Prevotella* | 0.50* |  |  |
|  |  | *Anaerococcus* | 0.43* |  |  |
|  |  | *Facklamia* | 0.40* |  |  |
|  |  | *Finegoldia* | 0.39* |  |  |
| **Complications** | |  |  |  |  |
| Yes | No | *Ezakiella* | 0.96* |  |  |
| **↑** | **↓** |  |  |  |  |

The table reports the consensus of the three differential abundance methods (LinDA, ALDEx2, and ANCOM-BC2) with their standardized effect sizes.

The * indicates adjusted *p*-value (Benjamini-Hochberg) < 0.05, ** indicates adjusted *p*-value (Benjamini-Hochberg) < 0.01, and when left empty values are not significant. N=sample size; direction of abundance, decrease **↓** increase **↑**

**Table S2** | **Clinical data of patients included in the study based on the development of complications after surgical intervention of PI**. Quantitative variables are expressed as median (Q1, Q3) and qualitative variables as absolute frequency (relative frequency in %). The nonparametric Mann-Whitney–Wilcoxon U test for independent variables was used to compare groups. For all measures, *p* < 0.05 was considered significant. Data analysis was performed with SPSS 25.0 (SPSS Inc., USA)

| **Variable** | **Complication after surgery of PI** | | | **U-test (p-value)** | |
| --- | --- | --- | --- | --- | --- |
|  | **Yes (n=8)** | | **No (n=19)** |  | |
| **Age** | 54 (47,58) | 59 (53, 66) | | | 0.20 |
| **Level of injury (thoracic)** | 4 (50%) | 13 (68%) | | | 0.48 |
| **Vertebra fractured (above T6)** | 4 (50%) | 11 (63%) | | | 0.62 |
| **ISNCSCI score (A)** | 6 (75%) | 12 (67%) | | | 0.66 |
| **Body mass index for SCI**  **(above 22.5 kg/m^2^)** | 6 (75%) | 10 (53%) | | | 0.39 |
| **Wound stage (IV)** | 3 (38%) | 10 (53%) | | | 0.55 |
| **Wound localization (Ischial)** | 6 (75%) | 12 (67%) | | | 0.66 |
| **Duration of wound (> 1 month)** | 5(77%) | 9 (47%) | | | 0.55 |
| **Area of wound (cm^2^)** | 38 (25, 54) | 20 (12, 28) | | | **0.029** |
| **Smoker (yes)** | 2 (25%) | 4 (21%) | | | 0.90 |
| **Prealbumin units (g/L)** | 0.21 (0.17, 0.28) | 0.24 (0.14, 0.28) | | | 0.94 |
| **Albumin (g/L)** | 35.5 (33.0, 38.5) | 36.0 (34.0, 38.0) | | | 0.84 |
| **CRP (mg/L)** | 20.5 (8.0, 62.0) | 10 (3.0, 52.0) | | | 0.39 |
| **Haematocrit (%)** | 34.5 (31.5, 37.5) | 34.0 (28.0, 40.0) | | | 0.98 |
| **Creatinine (µmol/L)** | 38.0 (36.0, 55.0) | 57.0 (48.0, 69.0) | | | **0.007** |
| **Preoperative osteomyelitis (yes)** | 3 (38%) | 2 (10%) | | | 0.28 |
